# Corrosion-Effected Bond Behavior between PVA-Fiber-Reinforced Concrete and Steel Rebar under Chloride Environment

**DOI:** 10.3390/ma16072666

**Published:** 2023-03-27

**Authors:** Xuhui Zhang, Xun Wu, Yang Wang

**Affiliations:** College of Civil Engineering, Xiangtan University, Xiangtan 411105, China

**Keywords:** polyvinyl alcohol fiber, bond degradation, corrosive damage, experimental study

## Abstract

Corrosion-effected bond behavior between polyvinyl-alcohol-fiber-reinforced concrete and steel rebar under a chloride environment is the experimental subject studied in the present work. Twenty-four pull-out specimens are designed and subjected firstly to an accelerated corrosion test. The effects of polyvinyl alcohol fibers on the cracking behavior, chloride penetration of concrete members and the corrosion loss of steel rebars during the corrosion test are discussed. After this, these corroded specimens are subjected to a pull-out test. The failure mode, the bond-slip curves and the typical bond-stress values are measured during the test. The effects of polyvinyl alcohol fibers and corrosion loss on bond behavior between polyvinyl-alcohol-fiber-reinforced concrete and steel rebar are clarified. Results show that the polyvinyl-alcohol-fiber-reinforced concrete exhibits worse resistance to corrosion damage than plain concrete. The cracking width, chloride penetration depth in concrete and the corrosion loss of steel rebar are more serious for the specimens with more polyvinyl alcohol fibers. The polyvinyl alcohol fibers also negatively affect bonding in ascending branches for both the specimens, but improve the bonding in descending branches after peak stress in the case of splitting. In the present test, the bond strength of corrosive specimens is increased slightly and then decreases gradually with the deepening of corrosion loss. The failures of specimens change from pull-out to splitting-pull-out as the corrosion time exceeds 30 days. Compared with uncorroded specimens, the maximum degradation of bond strength is about 50.1% when the corrosion is increased from 0% to 15%.

## 1. Introduction

Polyvinyl alcohol (PVA) fiber is a kind of organic synthetic material, which has many advantages compared with other fiber materials. PVA fiber has high strength and ductility with a relatively high elastic modulus [1], outstanding resistance to corrosion without toxicity, high hydrophilicity and tolerance of the alkaline environment in concrete, strong bonding with the cement matrix [2], low cost, and effective restriction of cracking of concrete over long term [2]. PVA fiber can substantially improve the post-cracking behavior of concrete, enhancing the ductility [3] and toughness [4]. It also increases the splitting tensile strength [5] and flexure strength [6]. Besides that, the frost resistance [7] and fatigue life of concrete are enhanced with the addition of PVA fiber [8,9].

At present, PVA fibers have been widely used in civil engineering such as in fiber-cement as asbestos replacement, Engineered Cementitious Composites (ECC), strengthening of enlarged section for concrete structures, and various kinds of shotcrete, which play a promising role in toughening and reducing cracking in concrete [10]. Meanwhile, some studies have been reported that the content of PVA fibers significantly affect the fluidity of concrete mixtures [11]. The flowability of concrete decreases obviously with the increase in PVA fibers, which will lead to increased porosity and micro-damage in the matrix [12]. In this case, PVA-fiber-reinforced concrete would be susceptible to environmental erosion, resulting in a serious durability problem. Some studies have been carried out on the durability [13,14] of PVA concrete, such as tests of its frost and permeability resistance [15]. Nowadays, however, very few works have been performed to clarify the corrosion-effected bond behavior between PVA-fiber-reinforced concrete and steel rebar subjected to a chloride environment.

Extensive works have been carried out to investigate the effect of corrosion on the bonding between concrete and rebar [16,17,18]. Goksu and Inci [19] found that the better mechanical interlock between slightly corroded rebar and surrounding concrete increased the bond strength between the rebar and surrounding concrete, as was also reported in the studies by Choi [20] and Kim [21]. However, for a higher degree of corrosion, the breaking down of the mechanically weak layers of the corrosion products with increasing corrosion, and the ductility of the structure, more rapidly decrease in this case [21,22]. Rakesh et al. [23] presented that the bond strength was enhanced prior to corrosion cracking and then rapidly reduced with increase in corrosion level. Khaled and Ted [24] studied the effect of corrosion on the bond between rebar and concrete; they found that the 15% corrosion loss in steel bar mass can decrease approximately 35.6% of the bond strength. The material properties and the mechanism of PVA-fiber-reinforced concrete are significantly different from those of plain concrete. It has been reported that PVA fiber increases the splitting tensile strength [25] and flexure strength [26,27] of concrete as compared with plain concrete. Besides that, the bridging effect of fibers in concrete result in a difference in the bonding mechanism. The bond behavior between the PVA-fiber-reinforced concrete and steel rebar corroded under a chloride environment is still not well understood.

In this paper, an experimental test is proposed to study the corrosion-effected bond behavior between PVA-fiber-reinforced concrete and steel rebar under a chloride environment. In the subsequent sections, the paper carries out work on the following parts. In Section 2, four specimens with different PVA fiber contents were designed and subjected to accelerated corrosion in chloride solution. The bond stress and slip of the specimens were tested by pull-out tests, and corrosion loss and chloride penetration depth were measured. In Section 3, chloride penetration depth, corrosion loss of steel rebars and corrosion-induced concrete cracking of specimens with different contents of PVA fibers are described. In Section 4, the effects of PVA fiber and corrosion loss on bond behavior between concrete and steel rebar are clarified. Based on the research, several conclusions are drawn in Section 5.

## 2. Experimental Program

### 2.1. Detail of Specimens

**Figure 1:**
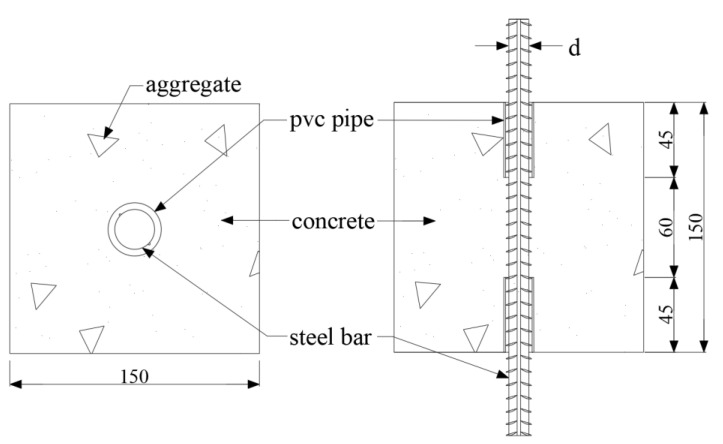
Detail of the specimen (Units: mm).

Twenty-four pull-out specimens were prepared in the present test, which were designed based on the Standard for Test Methods for Concrete Structures (GB 50152-1992) [28]. All the specimens were designed with the same size, which consisted of a 150 mm × 150 mm × 150 mm concrete cube in which a steel rebar was embedded, as shown in Figure 1. The diameter and the length of the steel rebar were 12 mm and 360 mm, respectively. The embedment length between the steel rebar and concrete was set as 60 mm for all the pull-out specimens, i.e., five times the diameter of the rebar, which was obtained by installing PVC tubes on both ends of the specimen to avoid the contact between concrete and rebar. The embedded length in the present work were designed based on the standard GB 50152-1992 [28] and similar experimental studies in the published papers [29,30,31].

The factors considered in the tests included four fiber volume contents (0%, 0.2%, 0.4% and 0.6%) and six salt solution immersive times (0 days, 10 days, 20 days, 30 days, 40 days and 50 days). Designation of specimens was coded with two parts. The first part represents the content of PVA fibers. The second part indicates the immersive times where 0 d, 10 d, 20 d, 30 d, 40 d and 50 d stand for immersive times of 0 days, 10 days, 20 days, 30 days, 40 days and 50 days, respectively. For example, PVA0.2-20d means that the fiber volume content was 0.2% and the immersive time was 20 days.

### 2.2. Materials and Mixture Design

The concrete was mixed using ordinary Portland cement, river sand, coarse aggregate, water, polycarboxylate superplasticizer and PVA fibers. The cement utilized in the test was ordinary Portland cement of 42.5 grade. The maximum coarse aggregate size was 20 mm with continuous graded crushed stone. The fine aggregate is natural river sand, and its fineness modulus is 1.82 by sieving test. PVA fibers produced by Kuraray in Tokyo, Japan were employed in the test. The diameter and length were 39 μm and 12 mm, respectively. The elastic modulus and the tensile strength of the fibers were 42 GPa and 1.6 GPa. The tested yield strength and ultimate tensile strength of the steel rebar were 454 and 521 MPa, respectively.

All specimens had the same mix proportions except the content of PVA fiber. The content of the cement was 485 kg/m^3^. The water–cement ratio was 0.42. The contents of coarse aggregate and fine aggregate were 1092 kg/m^3^ and 619 kg/m^3^, respectively. The content of the water-reducing agent was 0.8% by weight of cement. The content of PVA fiber was the percentage of concrete volume.

In the present test, the mixture was mixed with a forced mixer for 6 min. The fine and coarse aggregate were first dry mixed for 2 min. After that, water and water-reducing agent were pre-mixed and added into the mixture by 70% and mixed for 1 min. The PVA fibers were then dispersed gradually into the mixture and mixed thoroughly for 2 min. Finally, the remaining water and water-reducing agent were added the mixture, then mixed for another 1 min.

After the mixing procedure was completed, tests were conducted on the fresh mixture to determine the fluidity of the concrete. The slumps were 180 mm, 102 mm, 45 mm and 12 mm for concrete mixtures with 0%, 0.2%, 0.4% and 0.6% PVA fibers, respectively. It was found that the slump decreased by about 43%, 75% and 93% when the fibers were increased from 0% to 0.6%. This indicated that the PVA fiber obviously affected the fluidity of the concrete. This is consistent with the results reported in previous research studies [11]. This could be attributed to the roughness of the fiber surface, and the increase in the interface friction between cement and fiber.

Pull-out specimens and some reserved samples with the same content of PVA fibers were cast using the same mixing batches, which were demolded after 24 h and cured in the room at 20 ± 2 °C and 95% relative humidity. At the age of 28 days, the reserved samples were subjected to a mechanical property test and the pull-out specimens were subjected to the accelerated corrosion (see detail in the following section). The compressive strength, splitting tensile strength and flexural strength were tested according to GB/T 50081-2019 [32] and direct tensile strength tests were undertaken based on the method suggested by Dashti [33]. The mechanical property strengths of the samples with different PVA fiber contents and the coefficient of variation (Cv) are shown in Table 1. The coefficient of variation of the data obtained is less than 10%, which is weak variability, and thus the mechanical properties obtained below are accurate.

### 2.3. Accelerated Corrosion

**Figure 2:**
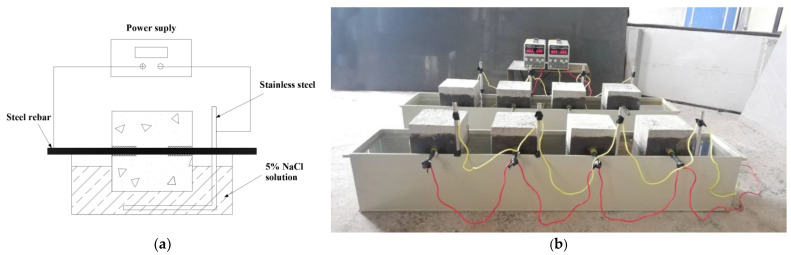
Setup of accelerated corrosion. (**a**) Setup device; (**b**) scene for the corrosion.

The electrochemical method was employed in the present study to accelerate the corrosion of specimens. Specimens were laid flat along the rebar on the corrosion tank and partially submersed in 5% sodium chloride solution. The stainless bar was connected with the negative terminal of the supply, and the steel rebar was connected with the positive terminal. The direct current flowed from the positive terminal to the rebar, and then through the saturated concrete and saline solution to the stainless bar, and finally back to the negative terminal. Figure 2 shows the setup of the accelerated corrosion system.

Before the accelerated corrosion test, specimens were partially submersed in sodium chloride solution for 3 days to saturate the concrete. The solution was kept approximately 10 mm below the rebar and the ends of the specimens were coated with anti-rust oil to prevent the permeability of chlorine salts along the rebar–concrete interface. A constant current was applied with the corrosion current density of 300 µA/cm^2^.

In this study, the corrosion current and pH of the solution were monitored and adjusted twice a day to ensure that they were in a stable state. To ensure the effectiveness of the liquid at a constant concentration, the solution was renewed at weekly intervals. Moreover, corrosion of rebars could induce the cracking of concrete covers. Therefore, the cracking on concrete surfaces was observed and measured to investigate the restraint of PVA fibers on corrosion-induced concrete cracking.

### 2.4. Pull-Out Test

**Figure 3:**
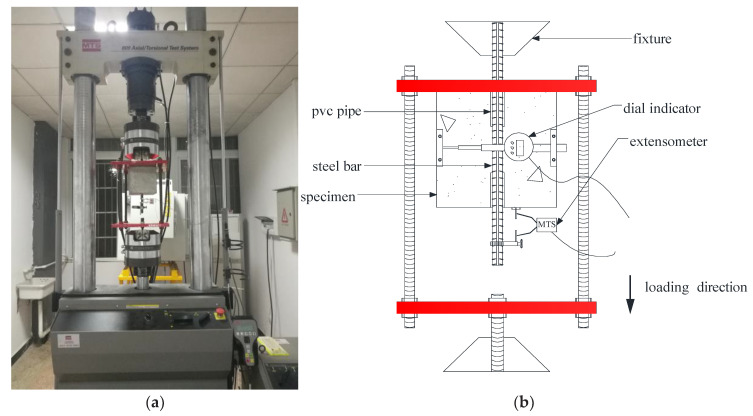
Loading instrument and detail device. (**a**) Loading instrument; (**b**) detail of device.

After accelerated corrosion, the specimens were subjected to a pull-out test, the test instrument is MTS-809 in the laboratory of Xiangtan University, the manufacturer of the instrument is the American MTS company (Monroe, NC, USA), and the maximum axial force capacity is 100 kN. A special loading frame was designed in the present test, as shown in Figure 3. The loading end of the specimens and the loading frame were fixed by the upper and lower clamps of the MTS, respectively. The position of the upper plate of the loading frame can be adjusted to fully contact with the specimen surface, which was used to minimize the effect of uneven stress and to prevent unexpected lateral movement of specimens during the loading process. The pull-out force was automatically measured by the test machine. The relative slip between the steel bar and concrete was measured by an extensometer clamped at the free end of the specimens. Additionally, a dial indicator was set transversely on the specimen surface to measure the development of the crack during the loading test.

The pull-out tests were monotonically loaded using a displacement-control mode at a speed of 1 mm/min. The experimental data, including pull-out force, relative slip and crack width were synchronously collected at the rate of 1 datum/s. The test was stopped as the splitting of specimens or the residual pull-out force trended to be constant. The measured slip between concrete and rebar at the free end was employed to represent the slip characteristic of the specimens. Additionally, the average bond stress along the embedded length of the rebar was used to represent the stress characteristic, which can be expressed as

(1) $$\tau =\frac{F}{\pi dL}$$

where *F* is the measured pull-out force; *d* is the nominal diameter of steel bar; and *L* is the embedded length of the steel bar in concrete. Bond stress and slip discussed in the following sections were obtained based on the above method.

### 2.5. Measurement of Corrosion Loss and Chloride Penetration Depth

After the pull-out tests, the specimen was split along the direction of rebar and the rebar was taken out to measure the actual corrosion loss. The rebars were cleaned with oxalic acid solution and then neutralized with alkali. After drying, the corrosion region, i.e., the bond region, of the steel rebar was cut off. The mass of the cut rebar was measured by using an electronic scale with the precision of 0.01 g. The actual mass loss of the corroded rebar was calculated as follows:

(2) $$\rho_{c}=\frac{m_{1}-m_{0}}{m_{1}}$$

where *ρ_c_* is the actual corrosion loss of rebar; *m*_1_ is the mass of the original rebar; *m*_0_ is the mass of the corroded rebar.

**Figure 4:**
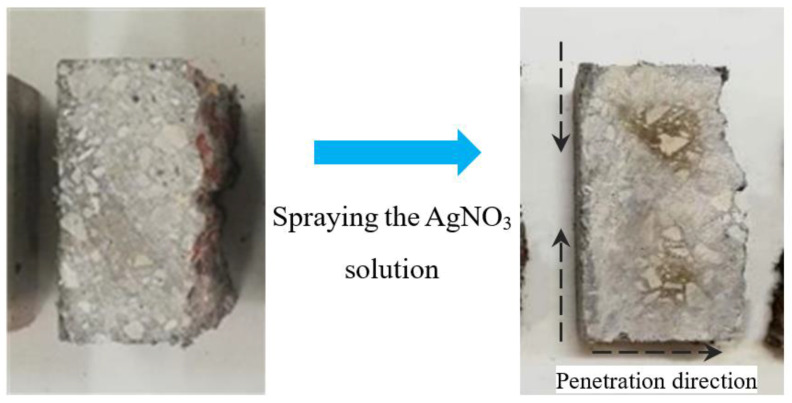
Color comparison.

Besides that, the split specimen was employed to measure the penetration depth of chloride in PVA-reinforced concrete based on the AgNO_3_ colorimetric method [34]. The split specimen was cut transversely in the middle, and AgNO_3_ solution with the concentration of 0.1 mol/L was evenly sprayed on the cutting faces of the concrete. The area penetrated by the chloride turned white, whereas the inner area turned brown, as shown in Figure 4, and the depth of chloride penetration was determined by the color difference.

**Figure 5:**
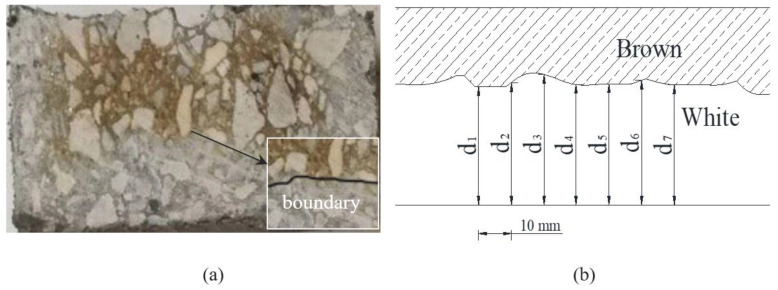
Discoloration border: (**a**) discoloration boundary; (**b**) illustration of chloride discoloration depth measurement.

According the recommendations of the NT Build 492 [35], the measurements of the depths of the white area were employed to represent the performance of chloride penetration, whereas the penetration depths was measured by using a slide caliper. From the center to both edges, tests were undertaken at intervals of 10 mm. Seven depths were measured and the average of the value was the chloride penetration depth, as shown in Figure 5b. The depth in the edge area of the specimen was not measured to avoid the edge effect caused by uneven saturation.

## 3. Corrosion Damages

### 3.1. Crack Behavior

**Figure 6:**
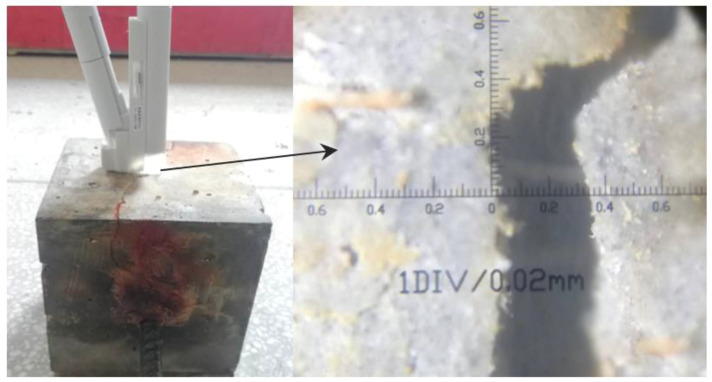
Crack observation.

In the corrosion process, the electrical potential applied to the positively charged steel bars attracted negatively charged chloride ions from the salt solution into the concrete. When the chloride ions permeated into the surface of the steel rebar, the bar began to corrode. However, corrosion deteriorated the steel ribs and filled the concrete–steel interface with rust products [36]. The expansive nature of the corrosion products could result in the initiation of a corrosive crack [37]. In these cases, the specimens were monitored with the method of periodic observation to determine the formation and propagation of corrosive cracks. The typical crack observation for specimens is shown in Figure 6.

**Figure 7:**
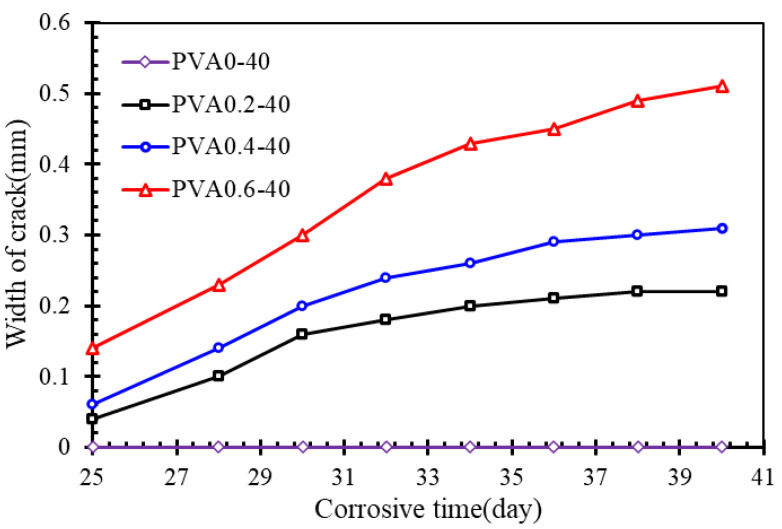
Crack width for different days.

Figure 7 shows the crack behavior of the PVA-12 series in different corrosive days. In particular, for the specimen with 0% PVA fibers, no sign of cracking was observed during the corrosion. The initial longitudinal crack along the direction of the rebar was observed on the bottom of the other specimens after about 25 days. The width of the initial crack increased obviously with the increment of PVA fibers, and after the corrosion time reached 30 days, the rising slope of the crack width growth curve also increased with the increase in PVA fiber. The slope of the ascending branch reflects the speed of rebar corrosion, which depends mainly on the resistance against chloride penetration for concrete. This indicates that the porous structure caused by addition of PVA fibers degrades the density of concrete. On the other hand, the growth rate of the crack width decreases gradually with the increase in the corrosion times. This could be attributed to the fact that the corrosion products flow out along the crack, which results in a reduction in the cycle of extrusion stress caused by corrosive products.

**Figure 8:**
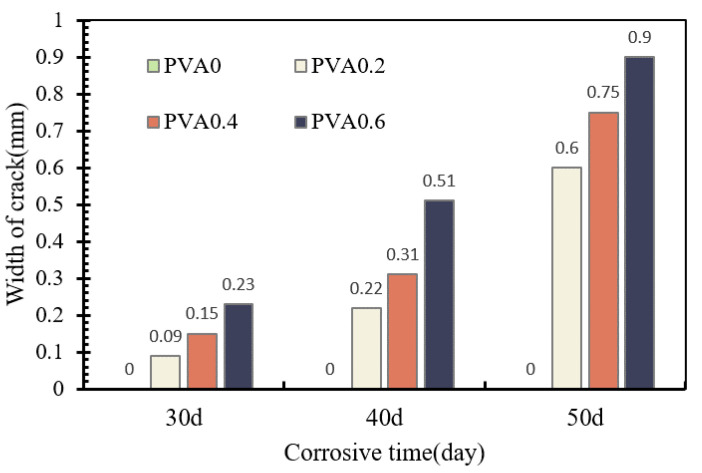
Maximum crack width.

Details of the maximum crack widths for corrosive specimens are shown in Figure 8. All the specimens show an incremental trend in crack width with the increase in corrosion times and PVA fibers. For instance, the mean maximum crack of specimens with 40 d and 50 d corrosion times was 2.24 and 5.19 times that of the specimen with a 30 d corrosion time, respectively. In this test, after adding PVA fiber, when the corrosion time reached 40 days and the PVA fiber content increased from 0.4% to 0.6%, the corrosion crack increment reached a maximum of 66.7%. Therefore, there is no positive influence of PVA fibers on corrosion cracking. As the corrosion level increases, the risk of corrosion of reinforcing steel within the concrete rises, which will harm the durability of the concrete. After the corrosion, the specimens were removed from the setup for visual inspection and pull-out tests.

### 3.2. Corrosion Loss

The corroded steel rebars were measured and the percentage of mass was computed using Equation (2), and the actual corrosion level is shown in Table 2. It can be seen that after the same number of corrosion days, there is significant difference in the corrosion degree of specimens with different fiber doping. The difference was mostly derived from the following three aspects. First, the permeability of the concrete with different PVA fiber contents was not included in the calculation of the theoretical level of corrosion. Although the specimens were immersed in the solution for three days prior to the accelerated corrosion, it would have taken a longer period for the chloride permeate to reach the surface of the steel rebar. Secondly, the incorporation of fibers increases the pore space of concrete, making the epoxy coating less protective than expected, which means that the corrosion range was larger than expected and resulted in a current density under 300 µA/cm^2^. Further, manual measuring errors during the weighting process of the rebar could also have caused the variation in corrosion loss.

**Figure 9:**
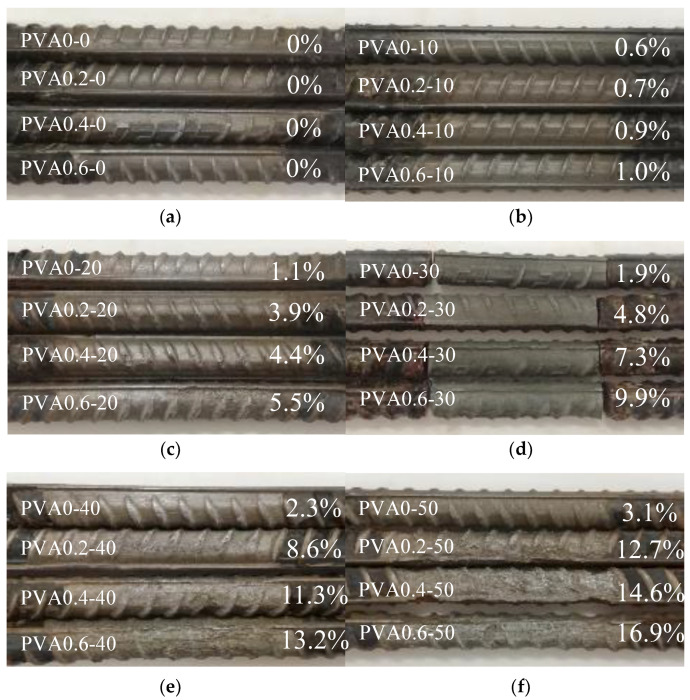
Appearance of corroded rebar: (**a**) 0%; (**b**) 3%; (**c**) 6%; (**d**) 9%; (**e**) 12% and (**f**) 15%.

Figure 9 shows the typical appearance of rebar with different corrosion levels. From top to bottom, the rebars were sequentially removed from the concrete with PVA fiber contents of 0%, 0.2%, 0.4% and 0.6%. The actual corrosion loss of the rebar was marked correspondingly. Compared with the appearance of the uncorroded rebar shown in Figure 9a, the rebar with lower corrosion loss, shown in Figure 9b,c, presented slightly concave corrosion pits, and the ribs coming out of the corrosion pits showed slight damage. At 9% and 12% corrosion levels, the area of the corrosion pits increased, accompanied by heavily damaged ribs, as shown in Figure 9d,e. At 15% corrosion level, the area of the corrosion pits increased further, as shown in Figure 9f, and the ribs had almost disappeared, resulting in a relatively smooth surface. In this case, the corrosive damage gradually deepened on the surface of the rebar with the increase in corrosion loss.

Based on the test results, the PVA-fiber-reinforced concrete specimens exhibited relatively worse corrosion resistance in the chlorine salt environment compared with the specimen with 0% fiber content. The more PVA fiber, the higher the corrosion loss of rebar with the same corrosive time. For example, the increase in PVA fiber volume content from 0% to 0.2%, 0.4% and 0.6% in the PVA-15 series specimens increased the corrosion loss by about 9.6%, 1.9% and 2.3%, respectively. The corrosion loss of the specimens without the addition of PVA fibers was usually lower in the present test, which may be the reason why corrosion cracking did not occur. Therefore, the addition of the PVA fibers led to smaller and more closely spaced cracks in the concrete, resulting in reduced permeability resistance of the concrete.

### 3.3. Chloride Penetration

**Figure 10:**
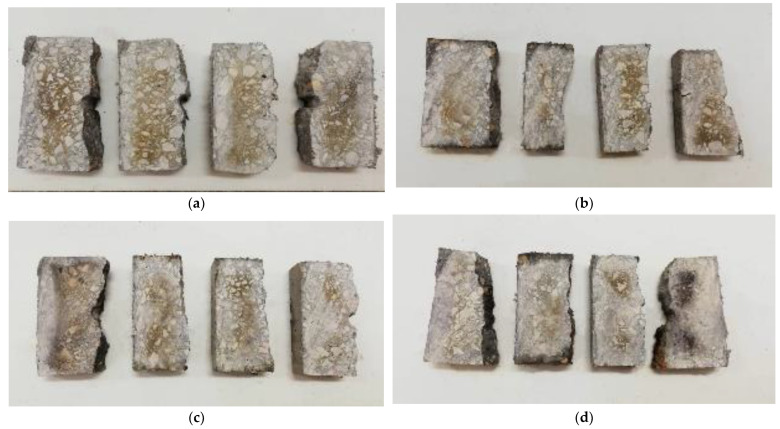
AgNO_3_ colorimetric results: (**a**) 20 d; (**b**) 30 d; (**c**) 40 d; (**d**) 50 d.

Figure 10 shows the colorimetric pictures of specimens with different contents of PVA fibers and salt solution immersive times. From left to right, the concrete sections were taken from the specimens with PVA fiber contents of 0%, 0.2%, 0.4% and 0.6%. As can be seen from Figure 10, the precipitated reaction products and color variation were clear on the concrete surface. The color change boundary was obviously different with the increase in immersive time. During the time of chloride penetration, all directions of the concrete were affected, which caused the white area to appear around the concrete cross-section. The color of part of the concrete surface became darker, which can be attributed to products of the oxidation reaction.

**Figure 11:**
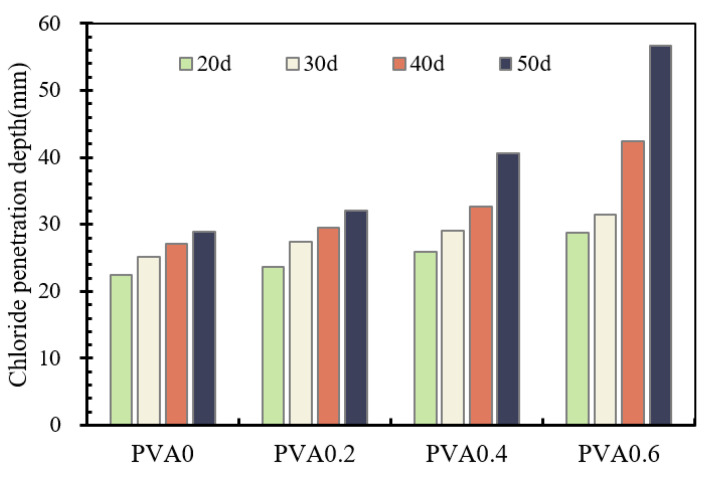
Chloride penetration depth.

In this test, the chloride penetration depths were measured from the side of the concrete. The color rendering within the boundary range of the concrete surface was not measured, to eliminate the influence of the boundary effect. The results of the depth measurement are summarized in Figure 11. Almost all of the chloride penetration depths are raised with the increase in immersive time. For example, the increase in corrosive time from 20 d to 30 d, 40 d and 50 d in the PVA0 series specimens increased the penetration depth by about 12.1%, 20.3% and 28.7%, respectively. The more the PVA fiber contents, the more obvious the penetration depth. For instance, the penetration depth of PVA0.2 series specimens increased by about 15.6%, 24.9% and 35.1% under the same corrosive conditions compared with the PVA0 series. It can also be noticed that when the same immersive times were used, incorporation of PVA fibers provided an additional penetrative path and increased the chloride penetration rate into the concrete, as compared with the concrete of the PVA0 series. The increase in depth was pronounced in the PVA0.6 series. In general, the addition of PVA fibers showed a negative effect on resistance to the chloride ion penetration.

**Figure 12:**
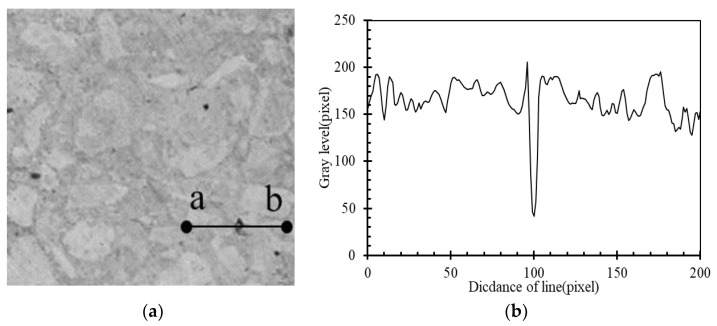
Detail of the specimen pieces and gray level. (**a**) Specimen pieces; (**b**) gray level.

After that, the concrete was cut into flat pieces, for which the area of 50 × 50 mm was selected for image processing to observe the tiny holes on the concrete surface through Image-J software. Firstly, the picture of the concrete pieces was gray processed as shown in Figure 12a. It can be seen that the concrete aggregate and mortar are lighter in color than the holes. Then arbitrarily determine a line segment that passes through a region with significantly higher grayscale values, such as the a-b line in Figure 12a. Then, the gray value along the a-b line segment, shown in Figure 12b, was determined. The curve shows that the gray value at the hole was significantly reduced. The gray level threshold of 130 was preliminarily selected in the present test and areas with a gray level below this threshold were identified as concrete pores.

**Figure 13:**
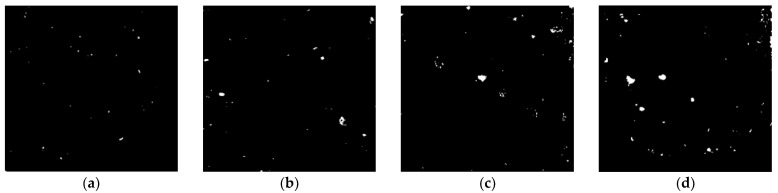
Binary processing image. (**a**) PVA0; (**b**) PVA0.2; (**c**) PVA0.4; (**d**) PVA0.6.

Figure 13 shows the binary processing image of concrete pieces in different PVA fiber volume contents. The number of pore structures on the surface of PVA0 series concrete was small, and the diameter of the holes was much smaller than that of the fiber concrete. The diameter and number of harmful holes were increased obviously with the increase in fiber contents. In this case, the incorporation of PVA fiber reduces the overall density of the concrete, which is consistent with the decrease in the compressive strength of fiber-reinforced concrete.

## 4. Bond Behaviors

Test results of the pull-out specimens, including the failure mode, the actual corrosion level *ρ_c_*, the bond stress at onset slip of free end *τ*_ons_, the maximum bond stress *τ*_max_ and the slip corresponding to the maximum stress *S*_0_ are summarized in Table 2. Because there is only one pull-out specimen per group, there is no coefficient of variation (Cv) here.

### 4.1. Failure of Pull-Out Specimens

**Figure 14:**
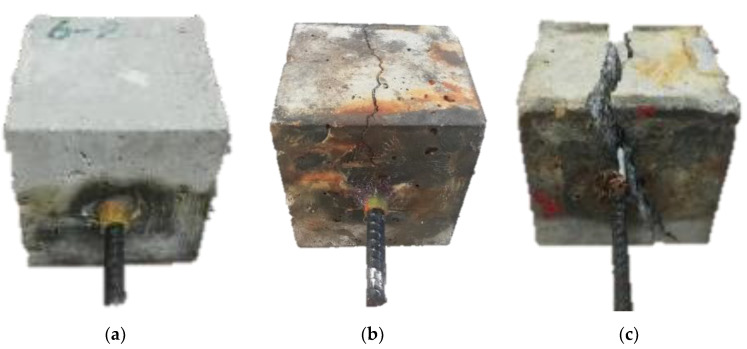
Typical failure of specimens: (**a**) pull-out failure; (**b**) splitting-pull-out failure and (**c**) splitting failure.

The specimens failed in three different modes: pull-out failure, splitting-pull-out failure and splitting failure. For the first one, the specimens failed gradually with the pull-out of the steel rebar without concrete cracking. For the second one, the specimens retained their integrity after the concrete splitting and the bond stress was not completely lost. For the third one, the specimens failed suddenly with the splitting of concrete accompanied by a loud crash. After that, the concrete members were completely broken apart. These three failure modes are shown in Figure 14.

Failure modes of all specimens are summarized in Table 2. It can be seen that the failure modes were affected by the corrosion level and PVA fiber volume contents. The specimens with 0 d, 10 d and 20 d corrosive times failed in pull-out mode due to the lower corrosion level and larger relative concrete cover depth. For the specimens of 30 d, 40 d and 50 d corrosive times, there was greater expansibility of corrosion products, with the result that the failure was splitting-pull-out mode. In particular, the specimen PVA0-50 failed in splitting mode. It could be attributed to the damage caused to the concrete and rebar by prolonged immersion in chloride solution. This indicates that the failure mode of the specimen tends towards splitting-pull-out damage as the corrosion time exceeds 30 days.

### 4.2. Effects of PVA Fibers

**Figure 15:**
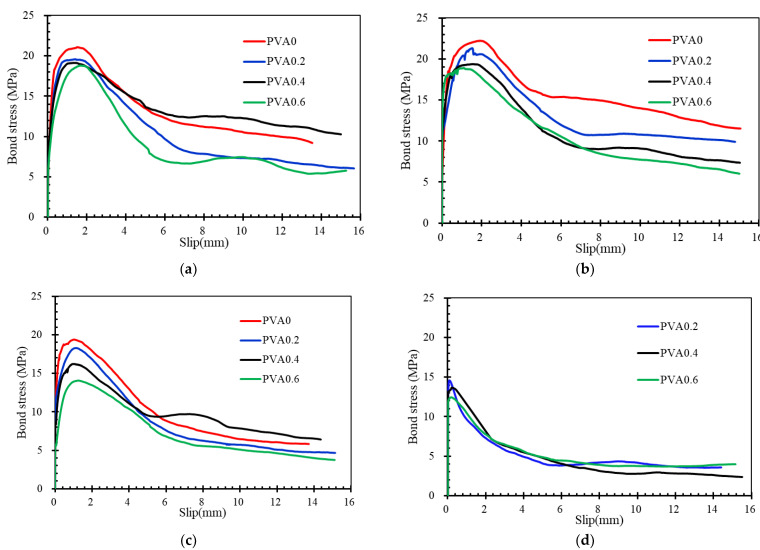

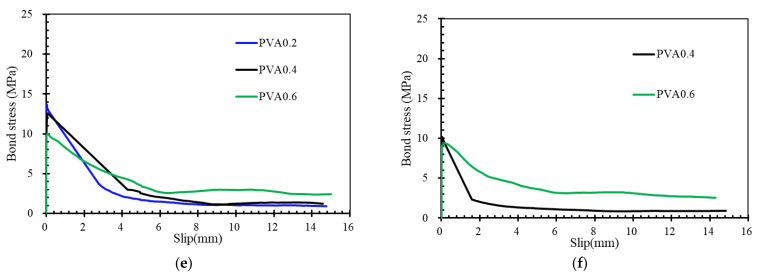
Bond stress–slip curves for the specimens with different PVA volume contents: (**a**) 0% series; (**b**) 1% series; (**c**) 5% series; (**d**) 8% series; (**e**) 12% series; (**f**) 15% series.

Figure 15 shows the bond stress–slip curves for specimens with different PVA fiber volume contents in the same rebar corrosion level. Since the corrosion degree of the steel bars in the PVA0 series specimens was generally low, this curve has been omitted from the comparison of the curves with a high corrosion degree, as shown in Figure 15d–f, below. As mentioned before, the curves are significantly affected by the failure modes. In addition, the additional PVA fibers also show an evident effect on bond stress–slip curves. The slope of the ascending branch of the bond stress–slip curve and the bond strength decreased with increasing PVA fibers for all specimens except for the 1% series specimen PVA0.6. The slope of the ascending branch reflects the bond stiffness of specimens, which depends mainly on the mechanical action between concrete and rebars. This indicates that the porous structure caused by the addition of PVA fibers degrades the mechanism between concrete and rebar.

The addition of PVA fibers shows a positive effect on the descending branches for specimens which failed in splitting-pull-out mode. After the curve has passed the peak load stress, the decline in bond stress can be effectively relieved with the increase in fiber content. Besides that, the residual stress was obvious for specimens with the addition of PVA fibers. The reasons should be attributed to the bridging effects of PVA fibers after the peak load stress of specimens, which effectively restrains the cracking and slows down the decrease in bond stress.

**Figure 16:**
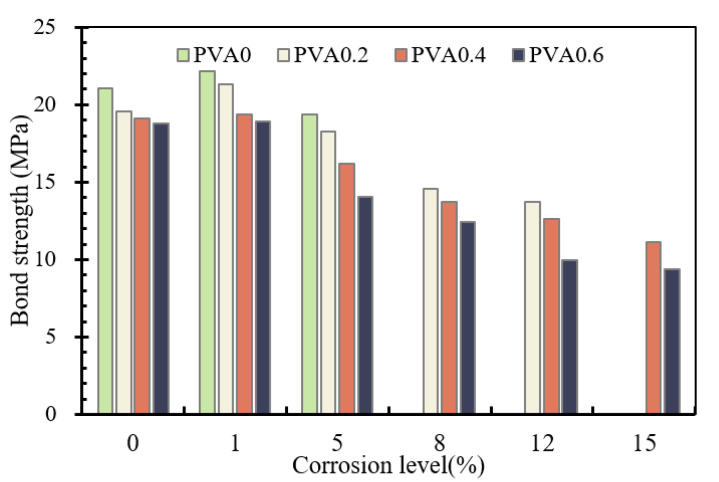
Bond strength of specimens with different contents of PVA fibers.

Details about the comparison of bond strength for specimens with different fiber volume contents are summarized in Figure 16. All the specimens show a descending trend in bond strength with the increase in PVA fiber. For example, the increase in PVA fiber volume content from 0% to 0.2%, 0.4% and 0.6% in specimens from the 1% corrosion level series decreased the bond strength by about 4.05%, 8.89% and 2.61%, respectively. The maximum decrement is about 20.85% in the present test.

**Figure 17:**
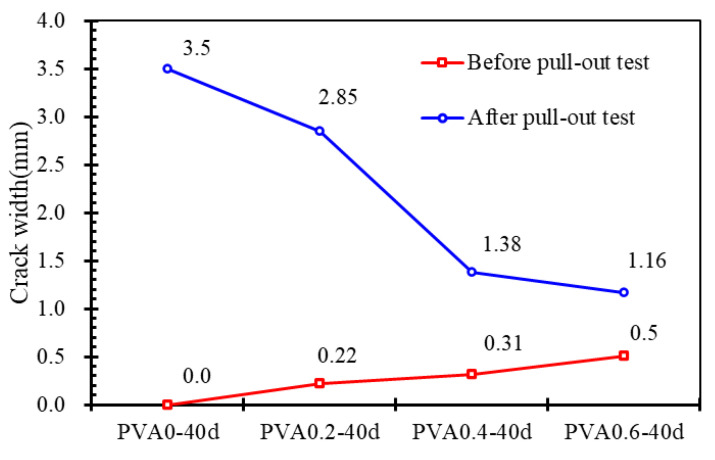
Crack width before and after pull-out test.

Figure 17 shows the comparison of crack width before and after the pull-out test for PVA-40 series specimens with different fiber volume contents. As mentioned before, the larger the volume content of PVA fibers, the larger the corrosion crack width of the concrete. However, in the pull-out test, it was found that the anti-drawing cracking property of the specimens gradually increased with the increase in PVA fiber content. Here, 0%, 0.2%, 0.4% and 0.6% of the PVA fibers caused the propagation of crack widths of about 3.5 mm, 2.6 mm, 1.1 mm and 0.7 mm, respectively. In this case, PVA fibers play a positive role in restricting the split-induced cracking. This indicates that PVA fibers restrict both the micro-cracking and macro-cracking of specimens which failed by splitting. The restriction effects on macro-cracking seem much more significant than that on micro-cracking.

### 4.3. Effects of Corrosion Loss

**Figure 18:**
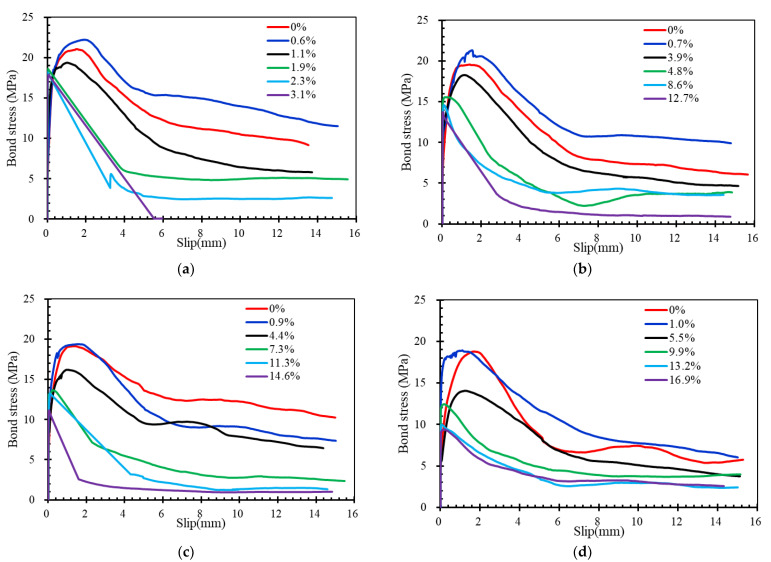
Bond stress–slip curves for the specimens with different corrosion loss: (**a**) PVA0 series; (**b**) PVA0.2 series; (**c**) PVA0.4 series; (**d**) PVA0.6 series.

Figure 18 shows the bond stress–slip curves for the specimens with different corrosion loss. The specimens in each subgraph have similar PVA fiber volume contents, but different corrosion levels. The curves match well with the failure modes for specimens with different corrosion levels. The curves show a gradual descent for specimens with low corrosion levels after the maximum bond stress, whereas there is an obvious rapid segment for specimens with high levels. Besides that, corrosion level also affects the initial bonding stiffness and bond strength of specimens. The initial bonding stiffness of each specimen usually increases gradually and then decreases with an augment in the corrosion of the steel rebar, except for individual specimens. The more the PVA fibers, the greater the difference of initial bond stiffness caused by different corrosion levels. In addition, the bond stress increases slightly at 1.0% corrosion loss. Specimens with high corrosion level have low bond stress.

The reason could be mainly attributed to the difference of the mechanical interlocking force between rebar and concrete. For cases with low corrosion levels, the positive effect was generated on bond properties because the slight corrosion of the steel bar improves the frictional resistance on the contact surface. However, with further increases in the corrosion level, the relative height of the ribs of the steel rebar gradually decreased. This caused the mechanical interlocking force between the concrete and the ribs of the rebar to significantly diminish. Besides that, cracking due to the volume expansion of the corrosion products reduced the confinement effect of the concrete. For the initial bonding stiffness of most specimens, however, the inflection point occurs at a higher corrosion level. The uneven corrosion of the rebar results in the corrosion damage on the upper surface being less than that on the lower surface. In this case, it could provide a certain friction at the beginning of the pull-out process. As the load increased, the friction was consumed and had no effect on the maximum bond stress. Therefore, the bond stress of all specimens initially increased slightly and then gradually decreased with further corrosion of the rebar.

**Figure 19:**
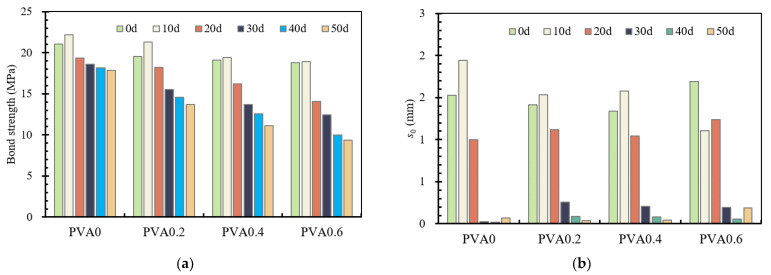
Bond stress–slip curves for the specimens with different corrosion loss. (**a**) Bond strength; (**b**) slip corresponding to bond strength.

Details about the comparison of bond strength and slip corresponding to bond strength for specimens with different corrosive times are summarized in Figure 19. As mentioned before, almost all of the bond strength and slip corresponding to bond strength increase with the increase in corrosive times. In particular, the bond strength and slip corresponding to bond strength increases slightly at low corrosive levels. For instance, the increase in corrosive time from 0 d to 10 d in PVA0.2 series specimens increased the bond strength by about 8.8% and the corresponding slip by about 8.4%. However, the increase in corrosive time from 10 d to 20 d, 30 d, 40 d and 50 d in specimens from the same series decreased the bond strength by about 14.3%, 14.8%, 6.3% and 5.8%, and decreased the corresponding slip by about 27.0%, 77.2%, 65.0% and 60.9%. Compared with uncorroded specimens, the maximum degradation of bond stress is about 50.1%, of which the corrosive time is 50 d. The more the PVA fibers, however, the greater the difference in the data, which is caused by corrosion loss. In general, the corrosion level of the rebar shows an obvious effect on bond strength and slip corresponding to bond strength for all specimens.

## 5. Conclusions

This study experimentally investigated the bond behavior between PVA-fiber-reinforced concrete and steel rebar corroded under a chloride environment. The effects of PVA fibers and corrosion loss on bond behavior were clarified. The following conclusions may be drawn based on the present study:

- The PVA-fiber-reinforced specimens exhibited worse resistance to corrosion damage than plain specimens; the harmful fine pores in fiber concrete provide channels for chloride penetration. The maximum increment of crack width is about 66.7% in the present test for PVA-fiber-reinforced specimens. With the increase in the fibers, the corrosive cracking become more obvious.
- PVA fiber generally showed a negative effect on bond behavior, but a positive effect on the descending branches for the case with splitting failure. PVA fibers decreased both the initial bond stiffness and bond strength in the present test. The maximum decrement of bond strength was about 31.49%, for samples with PVA fiber contents of less than 0.6%. The lowest extension of crack width was about 0.7 mm with the addition of PVA fibers in the pull-out test, in which the PVA fibers can restrict the split-induced cracking and protect against the failure of specimen in a more ductile way.
- With the deepening of corrosion loss, the bond strength of corrosion specimens first slightly increased, and then gradually decreased. Compared with plain specimens, the maximum degradation of bond stress was about 50.1%, for which the corrosion level was 15%. Specimens with a greater corrosion level usually had a greater initial bonding stiffness, but lower bond strength than specimens with a high level after uneven corrosion.
- There are no forces between the PVA-fiber-reinforced concrete and the reinforcement in the chloride environment explored in this paper. In engineering practice, PVA-fiber-reinforced concrete is often in the load-bearing state received under the influence of the external environment; the rust characteristics in the load-holding state remain to be further analyzed and studied.

## Figures and Tables

**Table 1:** Mechanical property strength of specimens.

Specimen Series	Fiber Content (%)	Compressive Strength/Cv (MPa/%)	Splitting Tensile Strength/Cv (MPa/%)	Flexural Strength/Cv (MPa/%)	Direct Tensile Strength/Cv (MPa/%)
PVA0	0	39.98/3	2.76/3	3.32/3	2.85/3
PVA0.2	0.2	38.21/3	3.35/3	3.48/2	3.00/2
PVA0.4	0.4	35.06/3	3.47/2	3.74/5	3.10/2
PVA0.6	0.6	37.23/5	3.69/2	3.81/2	3.25/4

**Table 2:** Experimental parameters and results of pull-out tests.

Test	Content of PVA Fiber (%)	*ρ_c_* (%)	*f_cu_* (MPa)	*f_t_* (MPa)	*f_ts_* (MPa)	*f_tf_* (MPa)	Failure Mode	*τ*_ons_ (MPa)	*τ*_max_ (MPa)	*S*_0_ (mm)
PVA0-0	0	0.0	39.98	2.85	2.76	3.32	P	4.95	21.08	1.53
PVA0.2-0	0.2	0.0	38.21	3.00	3.35	3.48	P	5.86	19.57	1.42
PVA0.4-0	0.4	0.0	35.06	3.10	3.47	3.74	P	6.10	19.10	1.34
PVA0.6-0	0.6	0.0	37.23	3.25	3.69	3.81	P	5.75	18.18	1.69
PVA0-10	0	0.6	39.98	2.85	2.76	3.32	P	6.36	22.20	1.94
PVA0.2-10	0.2	0.7	38.21	3.00	3.35	3.48	P	10.14	21.30	1.54
PVA0.4-10	0.4	0.9	35.06	3.10	3.47	3.74	P	10.40	19.41	1.58
PVA0.6-10	0.6	1.0	37.23	3.25	3.69	3.81	P	13.27	18.90	1.11
PVA0-20	0	1.1	39.98	2.85	2.76	3.32	P	10.64	19.38	1.00
PVA0.2-20	0.2	3.9	38.21	3.00	3.35	3.48	P	10.71	18.26	1.12
PVA0.4-20	0.4	4.4	35.06	3.10	3.47	3.74	P	8.65	16.20	1.04
PVA0.6-20	0.6	5.5	37.23	3.25	3.69	3.81	P	5.69	14.05	1.24
PVA0-30	0	1.9	39.98	2.85	2.76	3.32	S-P	17.19	18.60	0.02
PVA0.2-30	0.2	4.8	38.21	3.00	3.35	3.48	S-P	14.24	15.55	0.26
PVA0.4-30	0.4	7.3	35.06	3.10	3.47	3.74	S-P	13.13	13.70	0.21
PVA0.6-30	0.6	9.9	37.23	3.25	3.69	3.81	S-P	11.83	12.45	0.19
PVA0-40	0	2.3	39.98	2.85	2.76	3.32	S-P	18.00	18.17	0.02
PVA0.2-40	0.2	8.6	38.21	3.00	3.35	3.48	S-P	12.14	14.56	0.09
PVA0.4-40	0.4	11.3	35.06	3.10	3.47	3.74	S-P	9.55	12.59	0.08
PVA0.6-40	0.6	13.2	37.23	3.25	3.69	3.81	S-P	9.48	9.97	0.05
PVA0-50	0	3.1	39.98	2.85	2.76	3.32	S	16.98	17.88	0.07
PVA0.2-50	0.2	12.7	38.21	3.00	3.35	3.48	S-P	13.54	13.72	0.03
PVA0.4-50	0.4	14.6	35.06	3.10	3.47	3.74	S-P	9.94	10.13	0.04
PVA0.6-50	0.6	16.9	37.23	3.25	3.69	3.81	S-P	8.91	9.38	0.19

Notes: P means pull-out failure of specimens; S means splitting failure of specimens; S-P means splitting-pull-out failure of specimens.

## Data Availability

All data generated or analyzed during this study are included in this article.
